# Exosome-based engineering strategies for the diagnosis and treatment of oral and maxillofacial diseases

**DOI:** 10.1186/s12951-023-02277-4

**Published:** 2023-12-21

**Authors:** Jianing Ren, Xuan Jing, Yingyu Liu, Jinrong Liu, Xiao Ning, Mingrui Zong, Ran Zhang, Huaiyi Cheng, Jiayu Cui, Bing Li, Xiuping Wu

**Affiliations:** 1 Shanxi Medical University School and Hospital of Stomatology, Taiyuan, 030001 Shanxi China; 2 Shanxi Province Key Laboratory of Oral Diseases Prevention and New Materials, Taiyuan, 030001 Shanxi China

**Keywords:** Engineering, Exosomes, Oral and maxillofacial system, Diagnosis, Treatment

## Abstract

Oral and maxillofacial diseases are one of the most prevalent diseases in the world, which not only seriously affect the health of patients’ oral and maxillofacial tissues, but also bring serious economic and psychological burdens to patients. Therefore, oral and maxillofacial diseases require effective treatment. Traditional treatments have limited effects. In recent years, nature exosomes have attracted increasing attention due to their ability to diagnose and treat diseases. However, the application of nature exosomes is limited due to low yield, high impurities, lack of targeting, and high cost. Engineered exosomes can be endowed with better comprehensive therapeutic properties by modifying exosomes of parent cells or directly modifying exosomes, and biomaterial loading exosomes. Compared with natural exosomes, these engineered exosomes can achieve more effective diagnosis and treatment of oral and maxillary system diseases, and provide reference and guidance for clinical application. This paper reviews the engineering modification methods of exosomes and the application of engineered exosomes in oral and maxillofacial diseases and looks forward to future research directions.

**Graphical Abstract:**

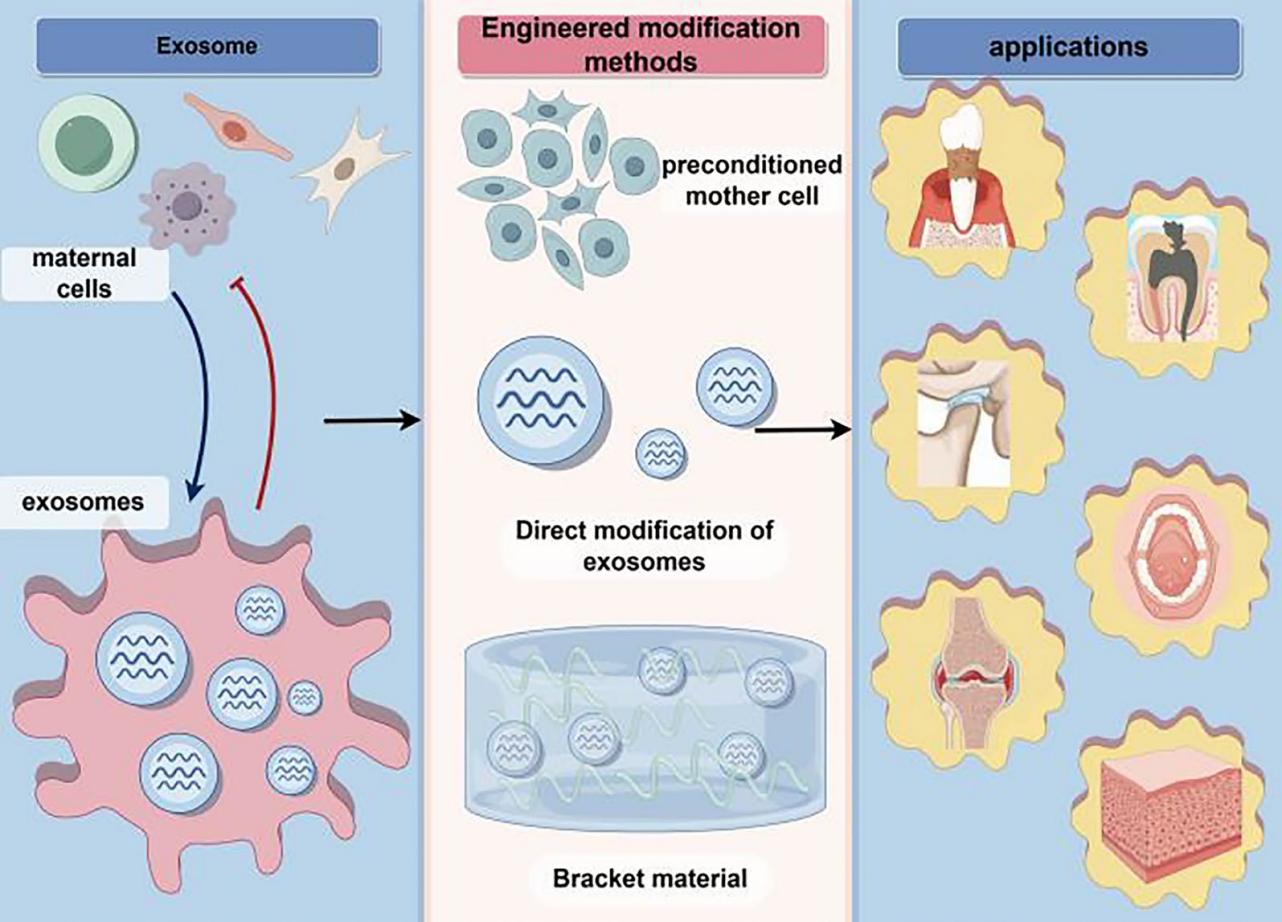

## Introduction

The oral and maxillofacial system is a complex physiological system with various anatomical structures such as teeth, periodontal tissues, jaws, mucous membranes, joints and other organs or tissues. Based on the above anatomical structures, the stomatognathic system has a variety of important functions including respiration, mastication, and speech. Oral and maxillofacial diseases are one of the most prevalent diseases in the world, which not only affect the physiological functions of the oral cavity but may even endanger the patients’ general health status, greatly reducing the quality of life of the affected individuals, and bringing a serious economic burden to the family and the society [1]. Typical oral and maxillofacial diseases, such as pulpitis, periodontitis, and oral cancer, often result in irreversible structural defects and associated dysfunction [2]. Therefore, effective methods are needed to treat oral and maxillofacial diseases. Traditional treatments have limited effects. In recent years, scholars have found that exosomes have a positive therapeutic role among the above mentioned diseases [3].

Exosomes were first proposed in the 1980s by Harding and Pan et al. [4, 5]. Exosomes are one of the extracellular vesicles (EVs) secreted by mother cells in the extracellular environment and are widely distributed in body fluids, which have a bilayered phospholipid membrane structure [6], exosomes contain large amounts of biologically active proteins, lipids, nucleic acids, etc. [7]. The functional status of exosomes-derived cells can be assessed by analyzing their exosomes contents, therefore exosomes can be used as one of the tools for disease diagnosis. Exosomes can also be involved in physiological processes by regulating the function of target cells through intercellular cargo molecular transfer, so they can also be used as a therapeutic tool for a variety of diseases, including tissue regeneration, immunomodulation, and cancer therapy [8, 9]. Currently, natural exosomes have been widely used in studies related to the treatment of oral and maxillofacial diseases, but the therapeutic effects of natural exosomes are unstable due to the complexity of their contents, lack of targeting, and ease of removal, and the underlying therapeutic mechanisms are complex and difficult to clearly explain [10]. By Engineered exosomes, the above drawbacks can be avoided appropriately and the diagnosis and treatment of oral and maxillofacial diseases can be achieved more efficiently.

## Biological characteristics of exosomes

**Fig. 1:**
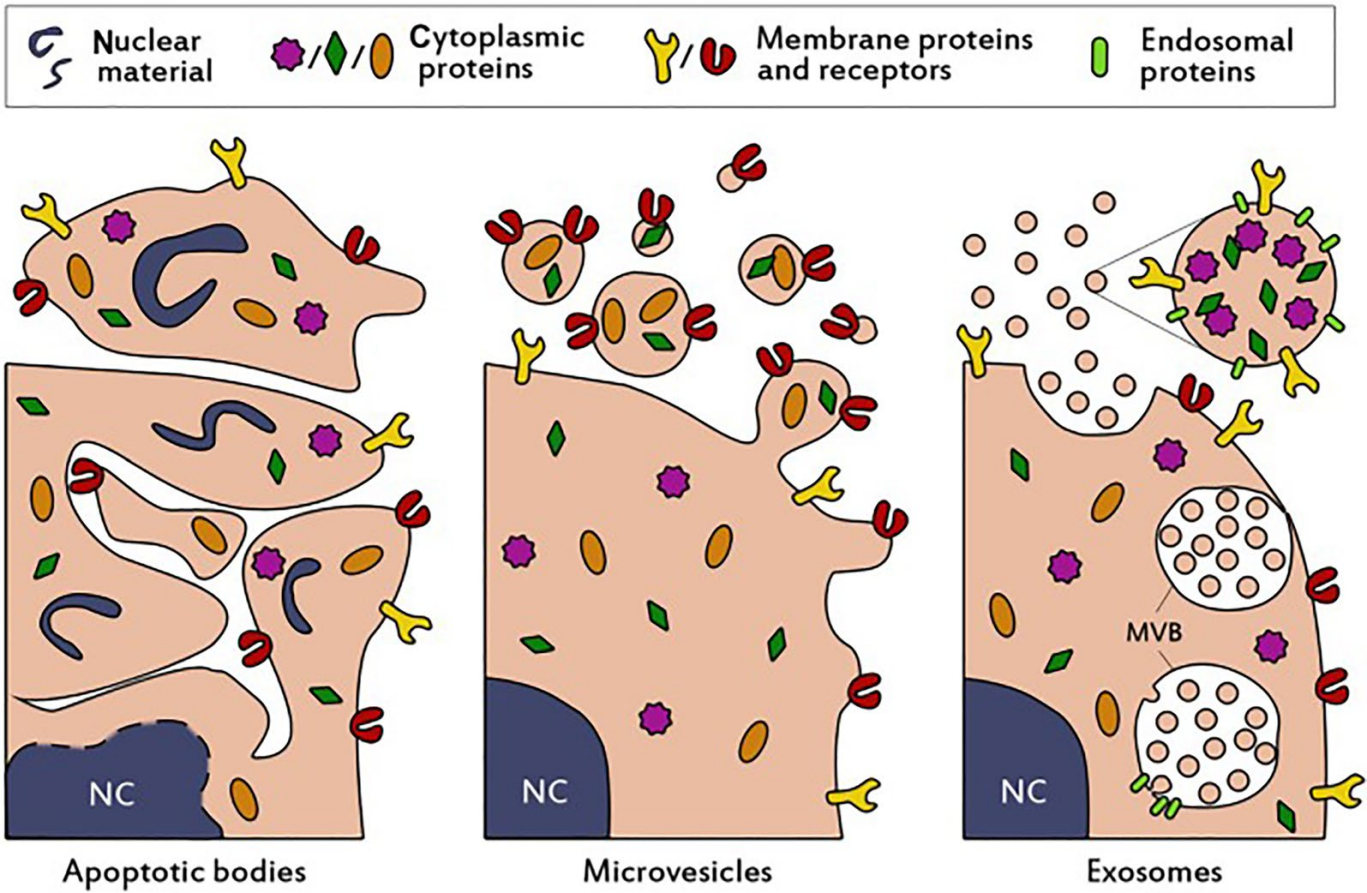
Schematic presentation of the biogenesis and composition of the three main classes of extracellular vesicles [22]

Exosomes are extracellular vesicles with unique production pathways and characteristics [11].Extracellular vesicles (EVs) are nanoscale vesicles produced by paracellular secretions [12].The formation process of EVs is first that the cell membrane of the mother cell sprouts inward through the “reverse budding” method to form early endosomes. Early nuclear endosomes are inwardly concave to produce multivesicular bodies; The multivesicular bodies finally fuse with the cell membrane under the action of Rab enzyme and excreted outside the cell by exocytosis, so the formation process of EVs can be simply summarized as budding-fusion-secretion [13, 14]. EVs can be classified into the following categories based on particle size: (1) apoptotic bodies (ApoBDs), with a diameter range of 500–1000 nm; (2) microvesicles (MVs), with a diameter range of 100–350 nm; and (3) exosomes (Exosomes, Exos), with a diameter range of 30–150 nm [15]. Figure 1 is a schematic diagram of the above categories. Lyden et al. further divided exosomes into three subgroups: large exosome vesicles (Exo-L, diameter 90–120 nm), small exosome vesicles (Exo-S, diameter 60–80 nm), and non-membrane nanoparticles (exomeres, diameter about 35 nm) [16]. Most cell types (mesenchymal stem cells, immune cells, cancer cells, etc.) can produce exosomes via the paracrine pathway, and exosomes are widely present in body fluids [17]. Exosomes are rich in a large number of biologically active substances such as proteins, DNA, mRNA and miRNA, which are similar to those of the parent cells. The level of the components of the exosomes mainly depends on the functional state of the parent cells. The analysis of changes in the content and composition of exosomes in the body fluids can reflect the state of the parent cells, which can provide a basis for the diagnosis of the oral and maxillofacial diseases and even the systemic diseases [18]. Moreover, exosomes can mediate intercellular signaling and participate in the development of diseases [19]. Exosomes carry specific protein markers (lactomucin, lysosome-associated membrane protein-2b (LAMP-2b), platelet-derived growth factor receptor (PDGFR), etc.), which makes exosomes have a strong homing and targeting capabilities [20, 21]. Nanoscale exosomes can effectively avoid phagocytosis by mononuclear macrophages and freely penetrate biological barriers to improve delivery efficiency; and due to their inherent biocompatibility, off-target effects can be reduced. Thus exosomes have natural drug delivery advantages and are promising drug delivery vehicles [22].

## Engineering strategies for exosomes

Although natural exosomes have been widely used in the treatment of oral and maxillofacial diseases, their shortcomings are still obvious, including low yield, high impurities, easy removal, and lack of targeting, lead to limited therapeutic efficacy of natural exosomes [23]. In recent years, it has been found that the use of exosomes for the precise treatment of oral and maxillofacial diseases is expected to be realized by engineering means [10]. The preparation methods of engineered exosomes include pretreatment of parent cells, direct treatment of exosomes, and biomaterials combined with exosomes, etc., and these methods can make exosome-related functions have obvious pertinence.

### Pre-treatment of parent cells

**Fig. 2:**
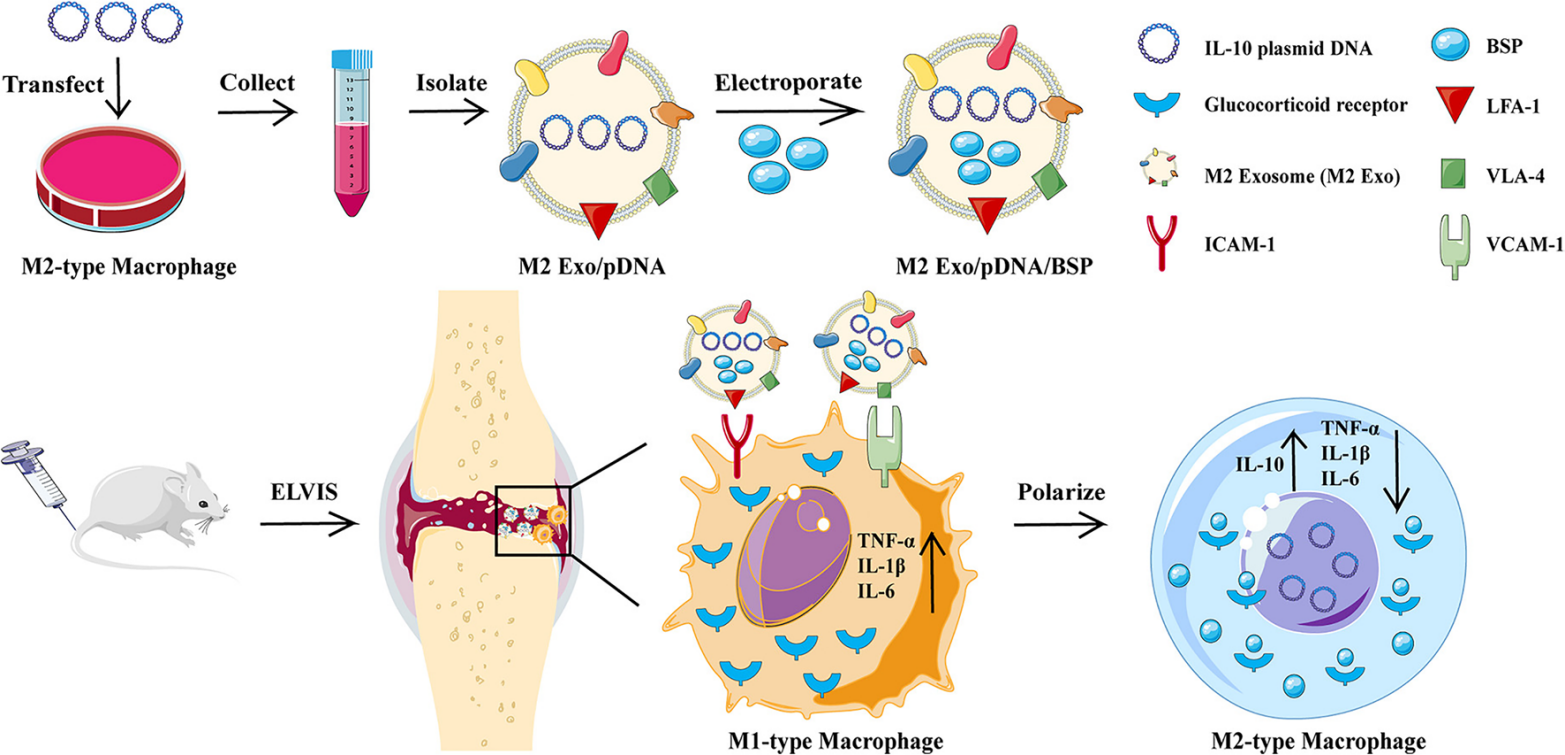
Engaged molecularly engineered M2 macrophage-derived exosomes with inflammation tropism and anti-inflammatory capabilities for co-delivery of IL-10 pDNA and GCs to achieve RA treatment via M1-to-M2 macrophages re-polarization [25]

Common methods of pre-treatment of maternal cells include transfection of maternal cells by lentivirus or plasmid to obtain exosomes carrying specific genes, pre-treatment of inflammatory factors, and pre-treatment of environmental conditions. It was found that synovial mesenchymal stem cells (SMSCs) could only promote fibroblast proliferation but not endothelial cell angiogenesis. Up-regulation of the expression level of exosome miR-126-3p produced by SMSCs by lentiviral transfection technique could promote both collagen production and angiogenesis, thus treating the prolonged diabetic wounds [24]. The imbalance between pro-inflammatory M1 and anti-inflammatory M2 macrophage activity in rheumatoid arthritis (RA) induces synovial inflammation and auto-immunity. Li et al. encapsulated plasmid DNA encoding the anti-inflammatory cytokine interleukin-10 (IL-10 pDNA) and the chemotherapeutic drug betamethasone sodium phosphate (BSP) in exosomes derived from M2-type macrophages, and found that the M2 Exo/pDNA/BSP cotransportation system promotes M2 macrophage polarity, which could be used as promising biocompatible drug carriers and anti-inflammatory agents in RA therapy [25]. The mechanism of macrophage repolarization in the treatment of rheumatoid arthritis is shown in Fig. 2.  Melatonin (MT)-pretreated MSCs-derived exosomes, MT-Exo, could promote diabetic wound healing by inhibiting inflammatory responses and increase M2 polarization to M1 polarization ratio by activating the PTEN/AKT signaling pathway. MT pretreatment proved to be a promising approach for the treatment of diabetic wound healing [26]. Atorvastatin (ATV), a HMG-CoA reductase inhibitor used to reduce the blood lipid in clinical. Exosomes (ATV-Exo) from bone marrow mesenchymal stem cells (BMSC) pretreated with ATV accelerate diabetic wound healing by enhancing angiogenesis [27]. Shi et al. found that 3,3ʹ-diindolylmethane-treated human umbilical cord-derived mesenchymal stem cells (HUCMSCs) could accelerate the healing of deep second-degree burn wounds by increasing the autocrine signaling of the exosome Wnt11 to promote the proliferation of HUCMSCs [28]. Wang et al. found that survival and proliferation of adipose stem cells were significantly enhanced after hypoxia induction, and the exosomes they produced could promote high-quality healing of diabetic wounds through PI3K/Akt pathway activation [29].

### Direct modification of exosomes

**Fig. 3:**
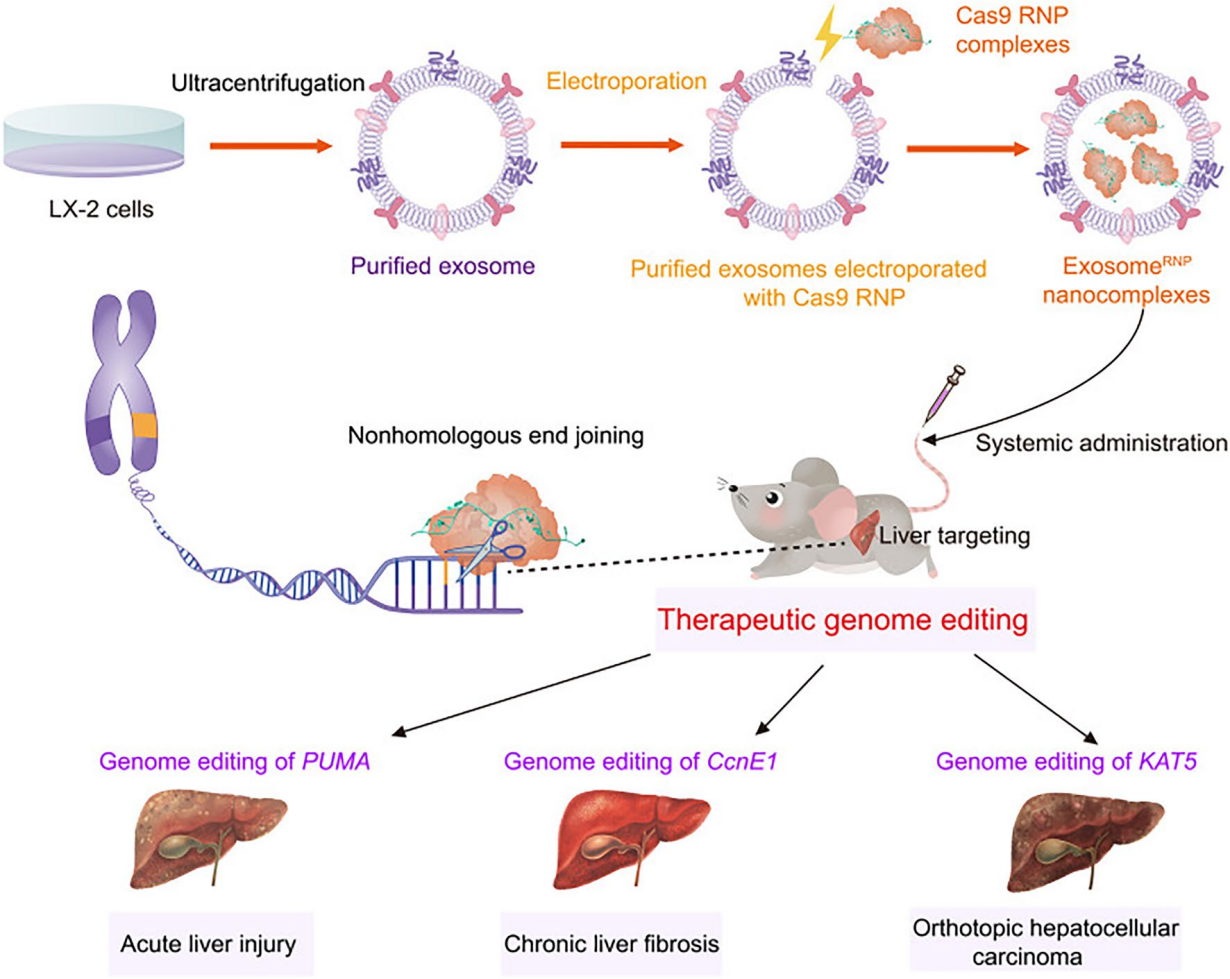
Schematic illustration of exosome for in vivo delivery of Cas9 RNP for the treatment of liver disorders [35]

Exosome modification has a more immediate and beneficial effect than pretreatment of parental cells. Exosome loading methods include co-incubation,ultrasound, and electroporation. Co-incubation is a commonly used method for direct modification of exosomes. Monocyte-derived myeloid cells play a crucial role in inflammation-associated autoimmune/inflammatory diseases and cancers, and EL-4 (mouse lymphoma cells)-derived exosomes are used by co-incubation with curcumin, which in turn enhances the therapeutic effect of exosomes on inflammation [30]. Early studies have reported that exogenous cargo can be loaded into exosomes via ultrasound [31]. Ultrasonic treatment maintains exosome stability and may facilitate cargo loading better than co-incubation [32]. Due to the lack of antimicrobial effect, exosomes are rarely used in the treatment of infected wounds. A study has conferred antimicrobial activity on exosomes derived from human umbilical cord mesenchymal stem cells (HUCMSC) by ultrasonically treating the exosomes to carry silver nanoparticles (AgNPs). An asymmetric wettable dressing is composed of the exosomes and the silver nanoparticle complexes, and TS-SF/SA/Ag-Exo Dressing has multifunctional properties that include broad-spectrum antimicrobial activity, promotion of wound healing, retention of moisture, and maintenance of electrolyte homeostasis [33]. Electroporation is another common method used to load exosomes with high cargo loading rates. At the same time, electroporation increases the amount of RNA and small hydrophilic molecules loaded in exosomes, thereby reducing RNA degradation in the wound microenvironment [34]. Tao et al. loaded Cas9 ribonucleoprotein (RNP) into purified exosomes isolated from hepatic stellate cells by electroporation, and found that this facilitated efficient cytoplasmic delivery of RNP while allowing RNP to accumulate specifically in liver tissue in vivo, which in turn facilitated tissue-specific gene therapy for liver disease, as depicted in Fig. 3 [35].

### Biomaterial loading exosomes

**Fig. 4:**
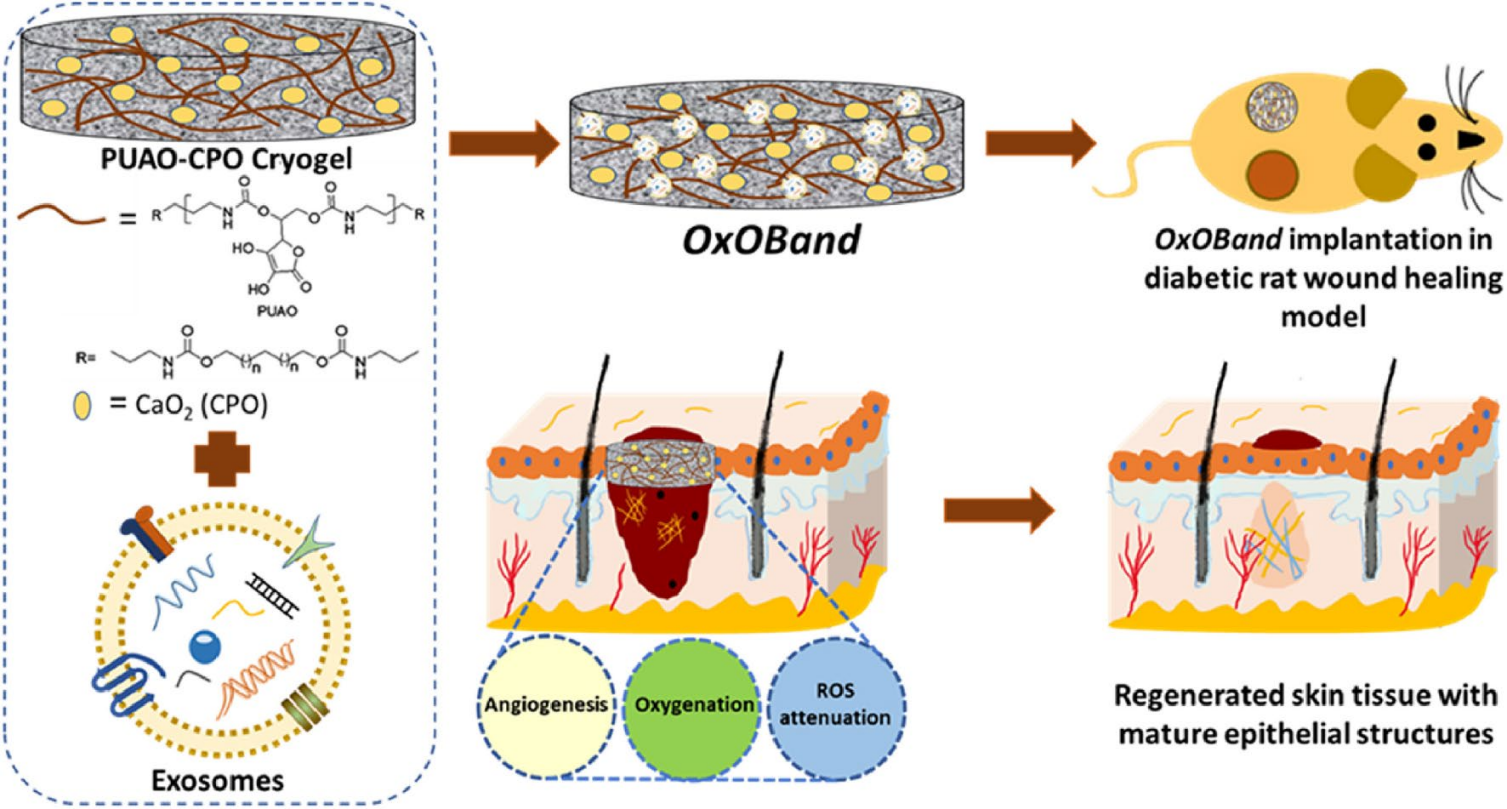
Schematic representation of the formation and application ofthe OxOBand [38]

Recent studies have reported that satisfactory therapeutic outcomes have been achieved through the use of various types of biomaterial examples (e.g. hydrogel scaffolds or dressings) to provide structural support and delivery of exosomes [36, 37]. This is shown in Fig. 4. A study has produced OxOBand, an exosome-rich, oxygen-releasing antioxidant wound dressing consisting of the antioxidant polyurethane (PUAO), a highly porous cryogel with sustained oxygen-releasing properties, loaded with adipose-derived stem cell (ADSC) exosomes. Experimental results show that OxOBand promotes faster wound closure, enhanced collagen deposition, faster re-epithelialization, increased angiogenesis and reduced oxidative stress within 2 weeks for enhanced diabetic wound healing and may lead to novel therapeutic interventions for the treatment of diabetic ulcers [38]. Jing et al. developed a bioresponsive polyethylene glycol (PEG)/DNA hybrid hydrogel and loaded it with human apical papilla stem cell-derived exosomes (SCAP-Exo), and found that the system was effective in promoting bone regeneration under both normal and diabetic conditions [39]. Cao et al. encapsulated umbilical cord mesenchymal stem cell (UCMSC) exosomes (UCMSC-Exo) in thiolated hyaluronic acid microgels and showed that they could promote osteoarthritis (OA) cartilage repair and rejuvenation of softened chondrocytes in a rat model [40].

## Diagnostic and therapeutic role of engineered exosomes in oral and maxillofacial diseases

### Periodontitis

Periodontal disease is a chronic inflammatory disease initiated by bacteria in plaque, such as Aggregatibacter actinomycetemcomitans and Porphyromonas gingivalis, invading the supporting tissues of the teeth (periodontal tissues), including two major types of gingival disease that only involves the gingival tissues, and periodontitis that involves the deeper periodontal tissues (periodontal ligament, alveolar bone, and cementum), which can lead to destruction of the supporting tissues of the periodontium, formation of periodontal pockets, loss of attachments, and resorption of alveolar bone, and with the progression of the disease, the tooth will slowly loosen and eventually lead to tooth loss [41]. It can affect 90 per cent of the global population [42]. Developing highly influential periodontal disease biomarkers is crucial for screening, diagnosis, and prediction of periodontal disease progression [43]. It has been demonstrated that engineered exosomes can be used to diagnose periodontitis. Studies have shown that salivary exosomes levels of CD9 and CD81 are significantly reduced in periodontitis patients compared to healthy controls [44]. Compared to healthy/gingivitis subjects, the total concentration of EV in gingival crevicular fluid (GCF) increased in periodontitis patients, and the CD63 exosome markers in GCF increased in periodontitis patients [45]. Exosome-based PD-L1 mRNA detection in saliva has the potential to differentiate between periodontitis and health, and its level correlates with the severity/stage of periodontitis [46]. Detection of miR-223-3p expression in salivary exosomes can be an important non-invasive method for diagnosing and assessing the severity of periodontitis [47]. Three significantly increased miRNAs (has-miR-140-5, hsa-miR-146a-5p and hsa-miR-628-5p) were detected in the salivary exosomes of patients with periodontitis compared to healthy controls, which had a good discriminatory power for the diagnosis of periodontitis [48]. Inflammatory periodontal ligament stem cells (PDLSCs) promote M1 macrophage polarization through miR-143-3p-mediated PI3K/AKT/NF-κB signaling regulation in their secreted exosomes. MiR-34c-5p inhibits osteogenic differentiation of PDLSCs via the SATB2/ERK pathway, providing a potential new target for periodontitis diagnose [49, 50].

**Fig. 5:**
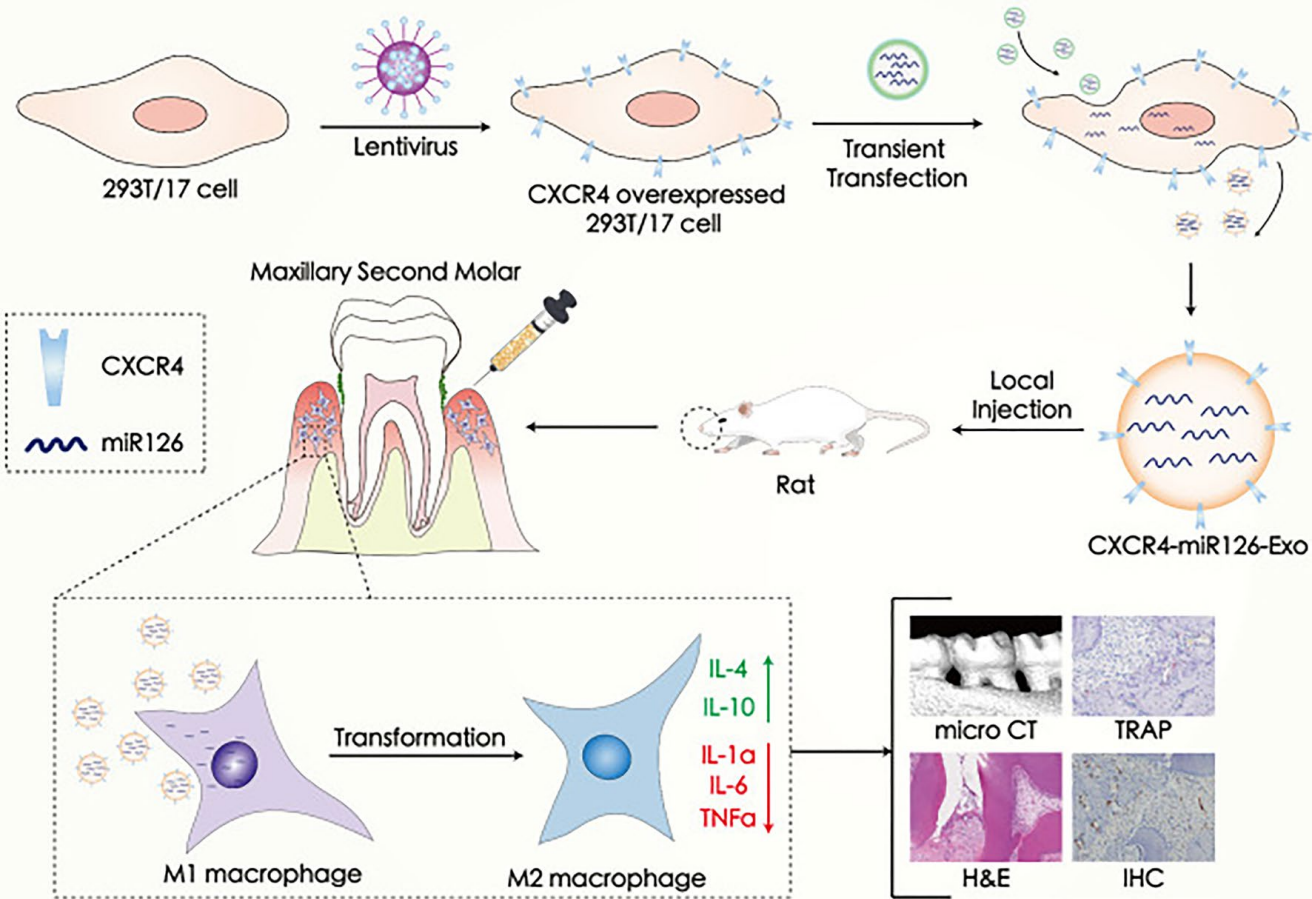
Schematic illustration of CXCR4-miR126-Exo production, targeting, and regulation of periodontitis in vivo [53]

Engineered exosomes have also been widely used to treat periodontitis. It has been shown that exosomes secreted by stem cells after P2X7 receptor(P2X7R) gene modification can exert a positive effect on their surrounding cells, and that P2X7R gene modification is able to reverse inflammation-mediated damage to periodontal ligament stem cells (PDLSCs) [51]. Exosomes from the inflammatory microenvironment enhanced osteogenic and odontogenic differentiation of PDLSCs in part by switching off LMBR1-targeted miR-758-5p via BMP signaling [52]. Figure 5 depicts exosomes overexpressing C-X-C motif chemokine receptor 4 (CXCR4) and loaded with miR-126 (CXCR4-miR126-Exo) reduced off-target delivery to macrophages and modulated the shift of macrophages towards an anti-inflammatory phenotype. In vivo local injection of CXCR4-miR126-Exo at the site of periodontitis in rats effectively reduced bone resorption and osteoclast formation and inhibited the progression of periodontitis [53]. It has been shown that exosomes released from human adipose-derived stem cells(hADSCs) delivered calcitonin gene-related peptide (CGRP) to human periodontal ligament stem cells (hPDLSCs), thus promoting the osteogenic differentiation potential of hPDLSCs. At the same time, the exosomes were also loaded into poly(lactic acid)-glycolic acid (PLGA) nanocomposite scaffolds grafted with hydroxyapatite (g-HA), and the constructed PLGA/pDA-EV system slowly released exosomes, which were implanted into the alveolar bone defect area of rats and significantly induced the repair of the bone defects [54]. The 3D culture system improved the functionality of MSC-Exo for the treatment of periodontitis, and 3D-Exo exhibited greater enrichment of miR-1246, which inhibits the expression of Nfat5 of Th17, and in turn treats periodontitis more effectively [55]. Shen et al. loaded the exosomes derived from dental pulp stem cells (DPSC-Exo) into chitosan hydrogel. The DPSC-Exo/CS system formed by Shen et al. promoted the conversion of macrophages from a pro-inflammatory phenotype to an anti-inflammatory phenotype in the periodontium of mice with periodontitis by a mechanism that may be related to miR-1246 in DPSC-Exo [56]. Collagen sponges loaded with MSC exosomes enhanced periodontal regeneration in a rat model of periodontal defects [57]. Chew et al. investigated the therapeutic effect of collagen sponges loaded with MSC exosomes in an immunocompetent rat model of periodontal defects, and found that MSC exosomes may enhance periodontal regeneration by increasing periodontal ligament cell carcasses and proliferation [49].

### Pulpitis

The dental pulp is rich in nerves and blood vessels, which respond to bacterial attack and injury by providing neuronal sensitivity and transmitting mechanical stimuli for repair and regeneration [58]. One of the most common endodontic diseases is pulp infection or pulp necrosis. Loss of pulp tissue can lead to lack of blood supply to the tissue of tooth, loss of tooth vitality, loss of hardness of the dental tissues, and lead to the disintegration and fragility of the teeth. The traditional treatment for endodontics is root canal therapy (RCT) [59].

A number of studies have been conducted to confirm that engineered exosomes are more effective than conventional therapies in the treatment of pulpitis. Human dental pulp stem cells (hDPSCs) released EVs in a mild inflammatory microenvironment were able to promote pulp regeneration through functional healing rather than scar healing [60]. Exosomes (Hypo-Exo) secreted from hypoxia-pretreated human deciduous papillary stem cells (SHED) promote angiogenesis by transferring let-7f-5p and miR-210-3p, as could be developed as a pro-angiogenic therapeutic strategy during pulp regeneration [61]. Exosomes derived from DPSCs and encapsulated exosomes overexpression of miR-125a-3p in exosomes promoted macrophage phenotypic shift to M2 type and enhanced BMP2 release from macrophages, which in turn promoted DPSCs-guided pulp regeneration [62]. Wang et al. developed a hydroxypropyl chitin (HPCH)/chitin whisker (CW) thermosensitive hydrogel with enhanced mechanical properties and bioactive. They embedded exosomes isolated from human dental pulp stem cells (hDPSCs) directly into HPCH/CW pre-gel to form exosome-loaded hydrogels (HPCH/CW/Exo), and experiments demonstrated that the delivery of exosomes significantly enhanced the hydrogel’s ability to promote pulp regeneration and angiogenesis [63]. Schwann cells (SCs) play a key role in the support, maintenance and regeneration of nerve fibers in the dental pulp. LPS may alter the intercellular signaling of hDPSCs, and exosomes secreted from hDPSCs after pretreatment with LPS promote the proliferation, migration and odontogenic differentiation of SCs, which may mediate the odontogenic differentiation of SCs [64].

### Temporomandibular disorders

Temporomandibular disorders (TMD) are a group of disorders involving the orofacial region, divided into those affecting the masticatory muscles and those affecting the temporomandibular joint (TMJ). Typical features include temporomandibular joint pain, limited jaw joint movement and temporomandibular joint sounds [65]. Engineered exosomes have also shown some efficacy in the treatment of TMD. Liu et al. found that inflammation-stimulated adipose-derived mesenchymal stem cell (ADSC)-derived exosomes (IAE) promoted cell proliferation and migration. Moreover, IAE significantly promoted M2 macrophage differentiation, and the high miR-27b-3p expression level in IAE may regulate macrophages by targeting macrophage colony-stimulating factor-1 (CSF-1). The TMJ condylar osteochondral defect model also showed that IAE significantly promoted TMJ regeneration [66]. Lee et al. successfully loaded molecules such as miR-140 into exosomes acting as an RNA delivery system by freeze-thawing and demonstrated the biologically active role of exosomes loaded with miR-140 in inducing the differentiation of BMSCs into chondrocytes and the facilitation of cartilage healing of the articular discs of the TMJ [67].

### Peri-implantitis

Peri-implantitis is a pathological condition that occurs in the tissues surrounding dental implants and is characterized by inflammation of the peri-implant mucosa and progressive loss of peri-implant bone tissue [68, 69]. Increased exosome concentration and downregulation of miRNA-21-3p and miRNA-150-5p expression may be associated with the development of peri-implantitis [70].

**Fig. 6:**
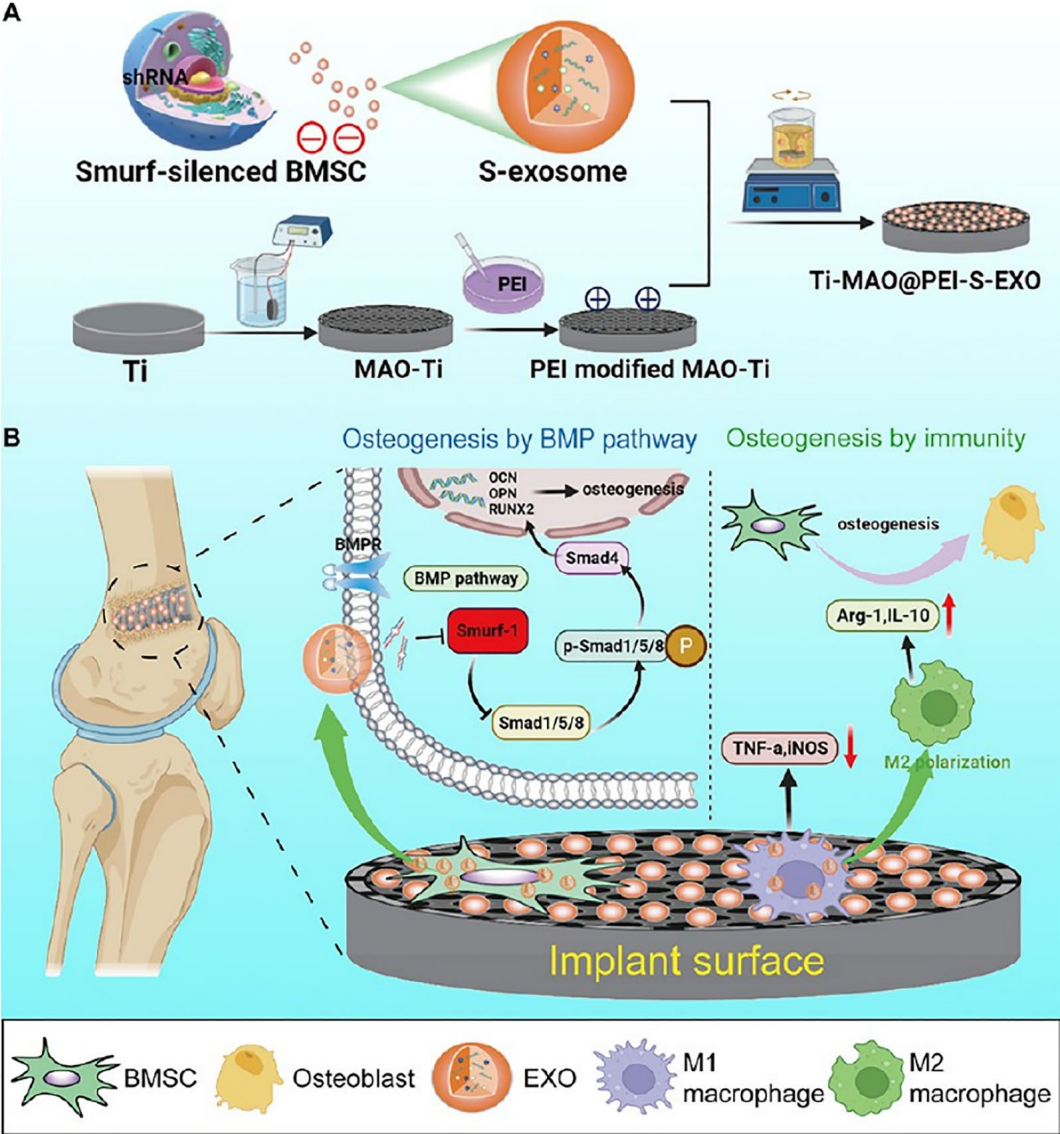
Schematic diagram of the construction of Ti-MAO@PEI-S EXO and its biological mechanism. **A** Preparation of Ti-MAO@PEI-S-EXO. **B** Mechanism of Ti-MAO@PEI-S-EXO to promote osteogenesis [73]

Engineered exosomes have shown promising effects on improving the peri-implant inflammatory environment and enhancing peri-implant osseointegration. Many clinical studies have focused on bone resorption of immediate implants, but few basic studies have been conducted on the mechanism between immediate implants and bone resorption. Wang et al. found that overexpression of ALKBH5 in MC3T3-E1-derived exosomes significantly rescued peri-implant bone loss, and that ALKBH5 acted on the circ_0008542 via demethylation, making circ_0008542 unsuitable for binding to the miR-185-5p/RANK axis. After reducing its molecular sponge effect, osteoclast differentiation and bone resorption around implants were also reduced. The potential value of this study lies in providing a way to enhance immediate implant resistance through the use of exosomes that release ALKBH5 [71]. The multistage titanium morphology of the micro/nanotube texture promotes the secretion of hBMSCs-derived exosomes through a cell proliferative effect and improves peri-implant osseointegration [72]. Xu et al. developed micro-arc oxidized titanium implants and loaded them with engineered exosomes (S-Exo) in order to promote osseointegration at the implant interface. They first transferred Smurf1-shRNA into BMSC using a viral vector to prepare S-Exo, which was subsequently immobilized on the surface of the micro-arc titanium oxide implant. The immobilized S-Exo could be released slowly and uniformly. The S-Exo subsequently phagocytosed by BMSC and macrophages, and this S-Exo coating exhibited the dual effects of promoting osseointegration and facilitating macrophage M2 polarization (Fig. 6) [73]. Li et al. constructed a fusion peptide (PEP) to act as a drug delivery system (DDS), exosomes derived from bone marrow mesenchymal stem cells (BMSC-Exo) have been shown to trigger osteogenic differentiation and mineralization of MSC. Both in vitro and in vivo experiments demonstrated that PEP retained the ability to bind both titanium and exosomes, and that the DDS gained the ability to target exosomes to the surface of titanium implants after enhancing post-implantation osseointegration, this Exo-PEP system could provide accurate and effective treatment for therapeutic implants [74].

### Sjogren’s syndrom

**Fig. 7:**
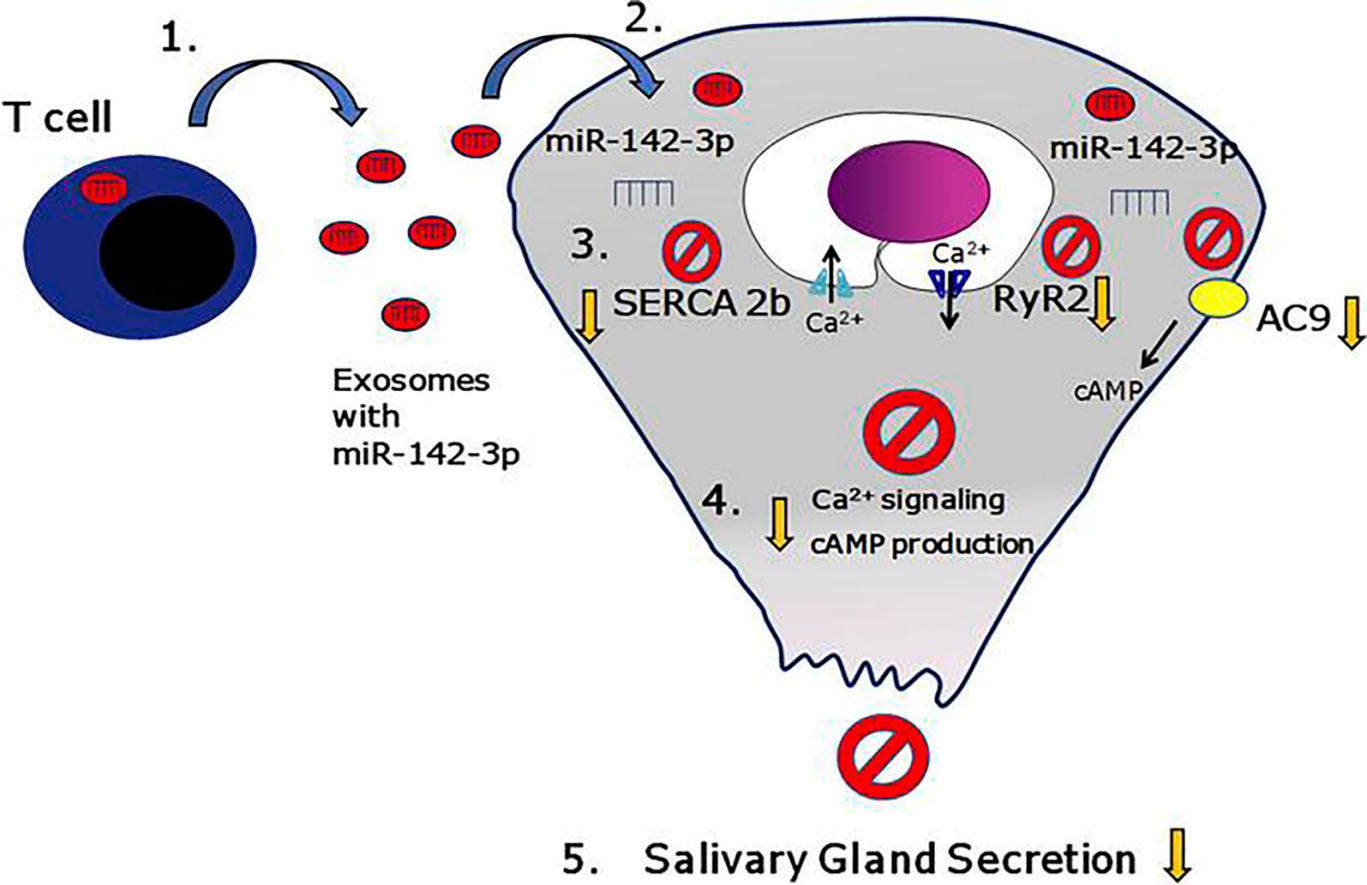
T cell exosome–derived miR-142-3p as a pathogenic driver of immunopathology in SS. MiR-142-3p is overexpressed in salivary gland lesions of SS patients [77]

Sjogren's Syndrom (SS) is a systemic chronic autoimmune disease of unknown etiology characterized by immune-mediated damage to the salivary and lacrimal glands, which results in dry mouth and dry eyes in patients. When evaluating patients with primary dry syndrom, non-invasive and more accurate diagnostic techniques are needed for a long time, and engineered exosomes can play this role. One study used liquid chromatography-mass spectrometry (LC–MS) to perform proteomic analyses of EVs isolated from saliva and tears of SS patients, and found that dozens of proteins were significantly up-regulated in the salivary EVs of SS patients compared to controls. Only 2 proteins were upregulated in the tears of SS patients due to the low volume of tears collected [75]. MiR-1290 and let-7b-5p have increased ratios that could serve as a novel and non-invasive diagnostic marker for SS [76]. Cortes-Troncoso et al. found that T-cells release exosomes containing a specific miRNA (miR-142-3p), miR-142-3p can target key proteins (SERCA2B, RyR2, AC9) that are critical components of the Ca^2+^ and cAMP pathways in salivary gland secretion [77]. The mechanism diagram is shown in Fig. 7. Therefore, an increased proportion of miR-142-3p may also serve as a novel and non-invasive diagnostic marker for SS.

### Oral lichen planus

Oral lichen planus (OLP) is a chronic inflammatory T-cell-mediated oral mucosal disease of unknown etiology. The main manifestations of OLP are white streaks, white papules, white plaques, erythema, erosion, or vesicles primarily involving the buccal mucosa, tongue, and gingiva [78]. Yang et al. found that OLP T-Exo induced increased expression of macrophage inflammatory protein (MIP)-1α/β, IL-10, and IL-17A thereby affecting cytokine secretion by T cells, and that MIP-1α/β may drive CD^8+^ T cell trafficking after binding to CCR1/5 in OLP, contributing to the development of OLP [79]. Thus OLP T-Exo may be used to diagnose OLP. MiR-4484 was significantly upregulated in salivary exosomes from patients with oral lichen planus [80]. Circulating plasma exosomes can also be used as potential diagnostic biomarkers for OLP. Peng et al. isolated exosome miRNA from the plasma of both OLP patients and healthy individuals, and by comparing them by miRNA array analysis they found that circulating exosome miR-34 a-5p was significantly upregulated in patients with OLP, and that exosome miR-34a-5p was positively correlation [81]. It is common for exosomes to play a diagnostic role in OLP, and there is still a lack of studies related to exosome therapy for OLP.

### Hard and soft tissue trauma defects of the maxillofacial region

Maxillofacial injuries are very common in Oral and maxillofacial diseases, and maxillofacial injuries usually trigger heavy bleeding because of the abundance of facial vascularization and the special location of the maxillofacial region, where the quality of healing and the speed of scarring healing are often of concern to the patient [82]. Engineered exosomes can promote the healing of hard and soft tissue trauma defects in the oral and maxillofacial region by modulating inflammation, improving angiogenesis, and promoting the proliferation and migration of tissue cells.

#### Engineered exosomes to regulate inflammation

In the early stages of wound healing, congestion, plasma exudate, leukocyte infiltration and localized redness are early signs of inflammation. In general, a mild inflammatory response is helpful because it helps to eliminate inflammatory factors, fight infection, and remove cellular debris, all of which help to regenerate injured tissue. In contrast, prolonged inflammation severely interferes with the wound healing process [83]. Su et al. infected the human melanoma cells (SK-MEL-5) with lentiviruses to get a stable cell line expressing human PD-L1. High concentrations of PD-L1 were obtained in secreted exosomes from genetically engineered cells overexpressing PD-L1 or stimulating MSC with IFN-γ. It was found that exosome PD-L1 binds specifically to PD-1 on the surface of T-cells and inhibits T-cell activation, thereby modulating the inflammatory response [84]. Exosomes from MSC stimulated with inflammatory factors such as tumor necrosis factor (TNF-α) and interferon (IFN-γ) have been found to reduce the release of pro-inflammatory cytokines and have the ability to improve the inflammatory environment [85]. It has been found that MSC-derived exosomes after LPS pretreatment have a better ability to regulate macrophage homeostasis as they upregulate the expression of anti-inflammatory cytokines and promote M2 macrophage activation through the let 7 b/TLR 4 pathway [86].

#### Engineered exosomes to improve angiogenesis

**Fig. 8:**
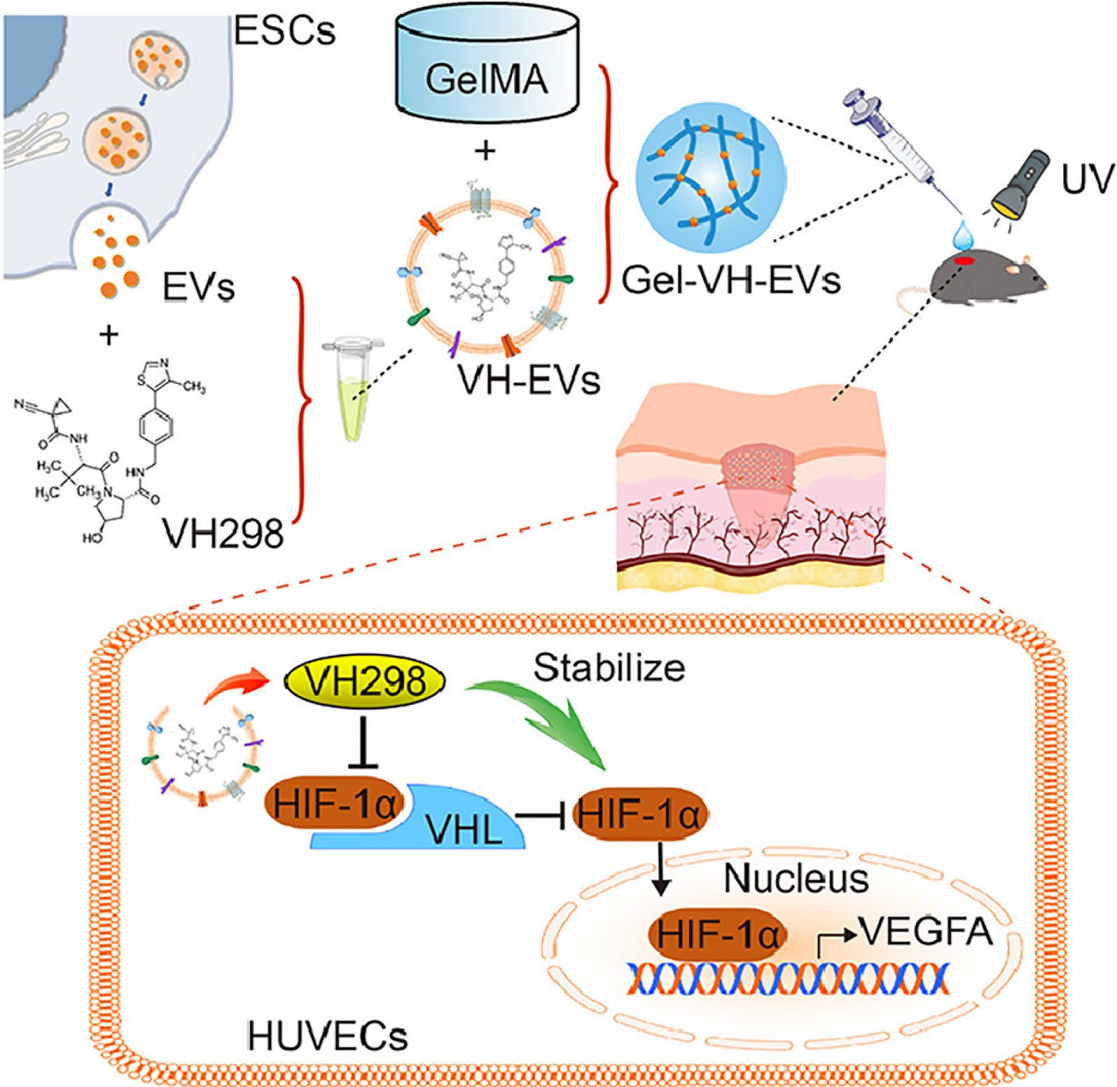
Schematic illustration of experimental procedure of the study and Underlying mechanisms of VH-EVs released from GelMA hydrogel for enhancing angiogenesis via stabilizing HIF-1α in HUVECs [90]

Angiogenesis is an intrinsic repair pathway for wound healing and tissue regeneration. Louis J Born et al. designed to observe the therapeutic potential of exosomes from mesenchymal stem cells (MSC) self-transfected to overexpress the long non-coding RNA HOX transcript antisense RNA (HOTAIR). HOTAIR has been found to be essential in mediating the angiogenic effects of endothelial cells, and MSCs were selected as exosome-generating cells for this study due to their widely reported intrinsic angiogenic properties. The experimental results showed that MSCs overexpressed HOTAIR (HOTAIR-MSC) produced exosomes with overexpression of HOTAIR. The HOTAIR-MSC-Exo promoted angiogenesis and wound healing in diabetic mice [87]. It was shown that superparamagnetic iron oxide NP-treated exosomes are precisely targeted and that they accumulate in damaged areas and significantly increase angiogenesis [88]. Synthetic exosomes with specific protein composition and RNA loading significantly promote angiogenesis [89]. Wang et al. loaded VH298 into epidermal stem cell (ESC)-derived exosomes and prepared photocrosslinked hydrogel gelatin methacryloyl (GelMA) containing VH-EVs (Gel-VH-EVs). Figure 8 shows the results revealed that VH298 improved angiogenesis by stabilizing HIF-1α and that Gel-VH-EVs had a significant therapeutic effect on skin defect repair [90].

#### Engineered exosomes to promote soft tissue wound healing

Fibroblasts play an important role in the healing of soft tissue wounds by synthesizing large amounts of collagen, whose orderly and adequate deposition is a key step in the proliferation and remodeling stages [91]. Wang et al. found that the survival and proliferation of adipose stem cells (ADSC) were significantly enhanced after hypoxia induction compared to anorexia. They also found that hypoxic adipose stem cell exosomes (HypADSC-Exo) can regulate the expression of various growth factors to promote fibroblast proliferation and migration, as well as angiogenesis. The HypADSC-Exo can accelerate diabetic wound healing by activating the PI3K/AKT pathway [29]. It has also been found that engineered stem cell exosomes carrying H19 can also increase fibroblast proliferation and inhibit apoptosis by affecting the H19/miR-152-3p/PTEN axis, which in turn regulates the PI3K/AKT signaling pathway, and ultimately promotes diabetic wound healing [92].

#### Engineered exosomes promote bone tissue regeneration

**Fig. 9:**
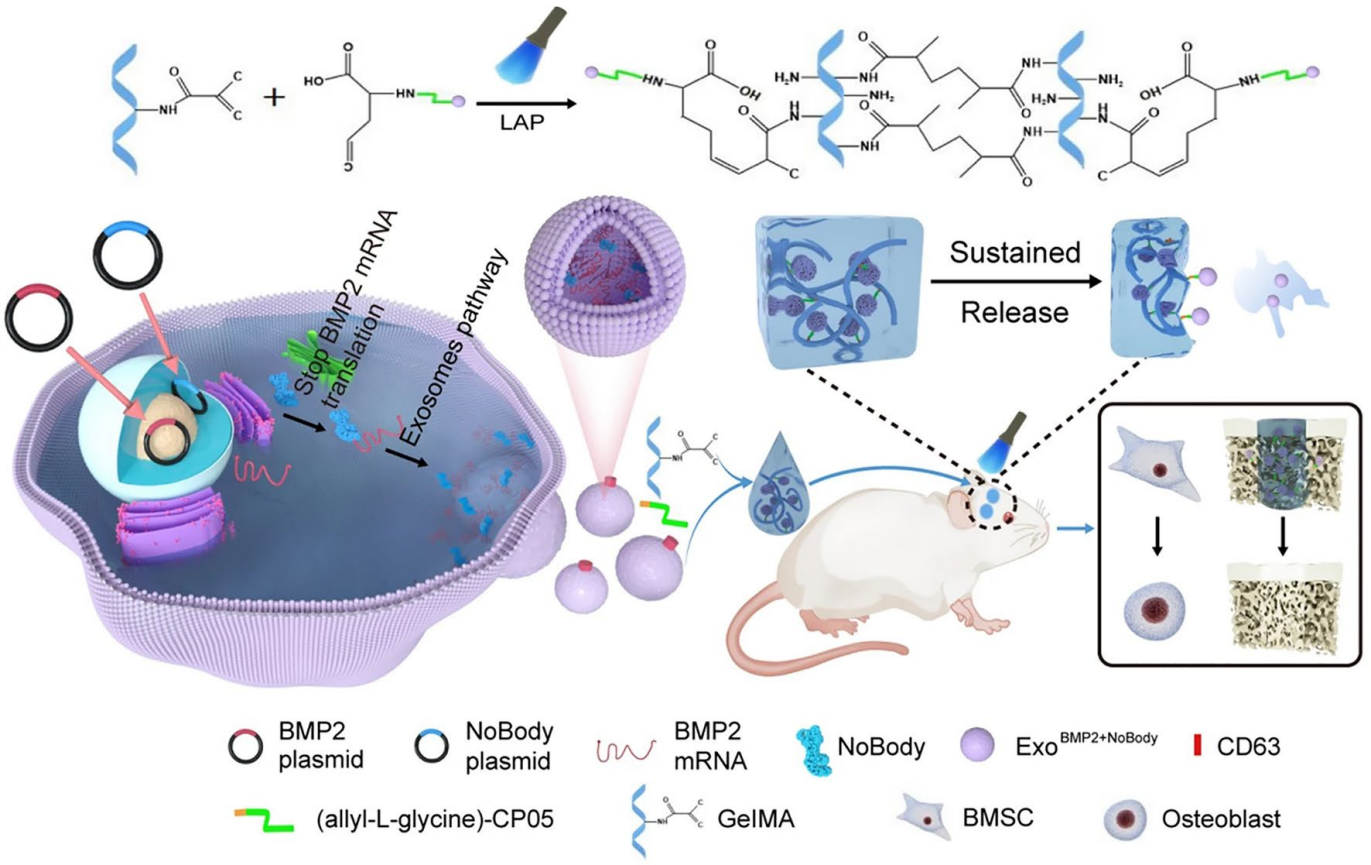
Schematical showing the preparation of ExoBMP2 + ^NoBody^-loaded GelMA and its effect on bone regeneration [94]

Engineered exosomes have also shown superior performance in promoting hard tissue regeneration. MiR-26a as an osteogenesis-related miRNA, Lai et al. extracted exosomes from the culture supernatant of miR-26a-modified BMSC by ultracentrifugation, and their experimental results revealed that miR-26a could be encapsulated into exosomes by DP7-C (a novel immunomodulatory peptide), and that exosomes loaded with miR-26a could promote osteogenesis and inhibit bone loss in experimental periodontitis and serve as a basis for new therapeutic strategies for periodontitis [93]. Figure 9 depicts the sustained release of bioactive bone morphogenetic protein-2 (BMP2) is important for bone regeneration, Yang et al. enriched the Bmp2 plasmid into BMSC-Exo by co-transfection, and the engineered exosome was loaded into GelMA hydrogel, and in an in vivo skull defect model, the ExoBMP2 + ^NoBody^-loaded GelMA showed a powerful ability to promote bone regeneration [94]. Dual functional regulation of angiogenesis and osteogenesis is essential for desired bone regeneration. Yao et al. developed a cell-free tissue engineering system that construct gene-activated engineered exosomes by using ATDC5 chondrogenic progenitor cell line-derived exosomes to encapsulate the VEGF gene. The specific exosome anchor peptide CP05 was used as a flexible connector to effectively combine engineered exosome nanoparticles with 3D-printed porous bone scaffolds, and engineered exosome-mediated bone scaffolds effectively induced mostly vascularized bone regeneration [95]. Liu et al. found that BMSCs-derived exosomes formed from Sr-CS-Exo after strontium-substituted calcium silicate (Sr-CS) stimulation had superior ability of promoting angiogenesis and osteogenesis [96].

### Oral cancer

Squamous cell carcinoma of the head and neck is the seventh most common malignancy worldwide with an annual incidence of > 600,000, about half of which are located in the oral cavity [97]. Squamous cell carcinoma arising from the oral mucosal epithelium is a fatal disease due to invasion of tumors, orofacial disruption, cervical lymph node metastasis and ultimately hematogenous dissemination. Oral cancer (OC) can be prevented and cured in its early stages. However, a significant number of OC cases are not diagnosed until the progressive stage, which is one of the major reasons for the poorer treatment responsiveness and prognosis [98]. Patients with oral cancer are at high risk of secondary cancers, and there are no available biomarkers to detect them until the patient develops a visible lesion and is diagnosed by biopsy. Exosomes secreted by cancer cells are involved in growth of tumors, invasion and metastasis, the use of exosomes is a very promising biomarker for the diagnosis of oral cancer. OC show a different expression of exosome markers: lower expression of CD81 and CD9 and higher expression of CD63 [99]. Salivary exosomes of oral and oropharyngeal squamous cell carcinomas showed elevated expression of miR-486-5p, miR-1307-5p, miR-10b, miR-130a, miR-210, and miR-365, and decreased expression of miR-10b-5p, compared to healthy controls [100–104]. While miR-512-3p and miR-412-3p were upregulated in salivary exosome from oral squamous cell carcinoma (OSCC) patients, miR-302b-3p and miR-517b-3p were expressed only in exosome from OSCC patients [105]. Salivary exosome miR-24-3p maintains OSCC cell proliferation by targeting PER1, miR-24-3p is a potential novel diagnostic biomarker for OSCC [106]. Has_circ_0069313 is an exosome circRNA, has_circ_0069313 induced oral squamous cell immune escape via the miR-325-3p-Foxp3 axis in oral squamous cells and Treg cells, has_circ_0069313 is up-regulated in oral squamous cell carcinoma tissues [107]. Deng et al. used a combined strategy of microarray of exosome circRNAs and qRT-PCR validation, found that three types of circRNAs from OSCC were associated with the risk of preoperative lymph node metastasis (LNM) risk, including hsa_circRNA_047733, hsa_circRNA_024144, and hsa_circRNA_403472. They also demonstrated that hsa_circRNA_047733 may be a novel biomarker for LNM in OSCC [108]. Circulating PD-L1 on the surface of exosomes isolated from plasma emerged as useful indicators of disease and immune activity in patients with head and neck squamous cell carcinoma (HNSCC) [109]. OSCC LN1-1 cells showed greater capacity for lymphatic genesis and lymph node metastasis than their parental OEC-M1 cells, in addition to the ability to enhance migration and tube formation of lymphatic endothelial cells (LECs). Uptake of laminin γ2-rich exosomes by LECs enhanced the formation of lymphatic vessels in vitro, and thus laminin-332-carrying exosomes are a viable biomarker for OSCC [110]. One study examined serum exosomes (SE) in OSCC patients with lymph node metastases (LNM), and they concluded that PF4V1, CXCL7, F13A1, and ApoA1 in SE may be associated with OSCC metastasis, which could be helpful in the diagnosis of OSCC [111].

**Fig. 10:**
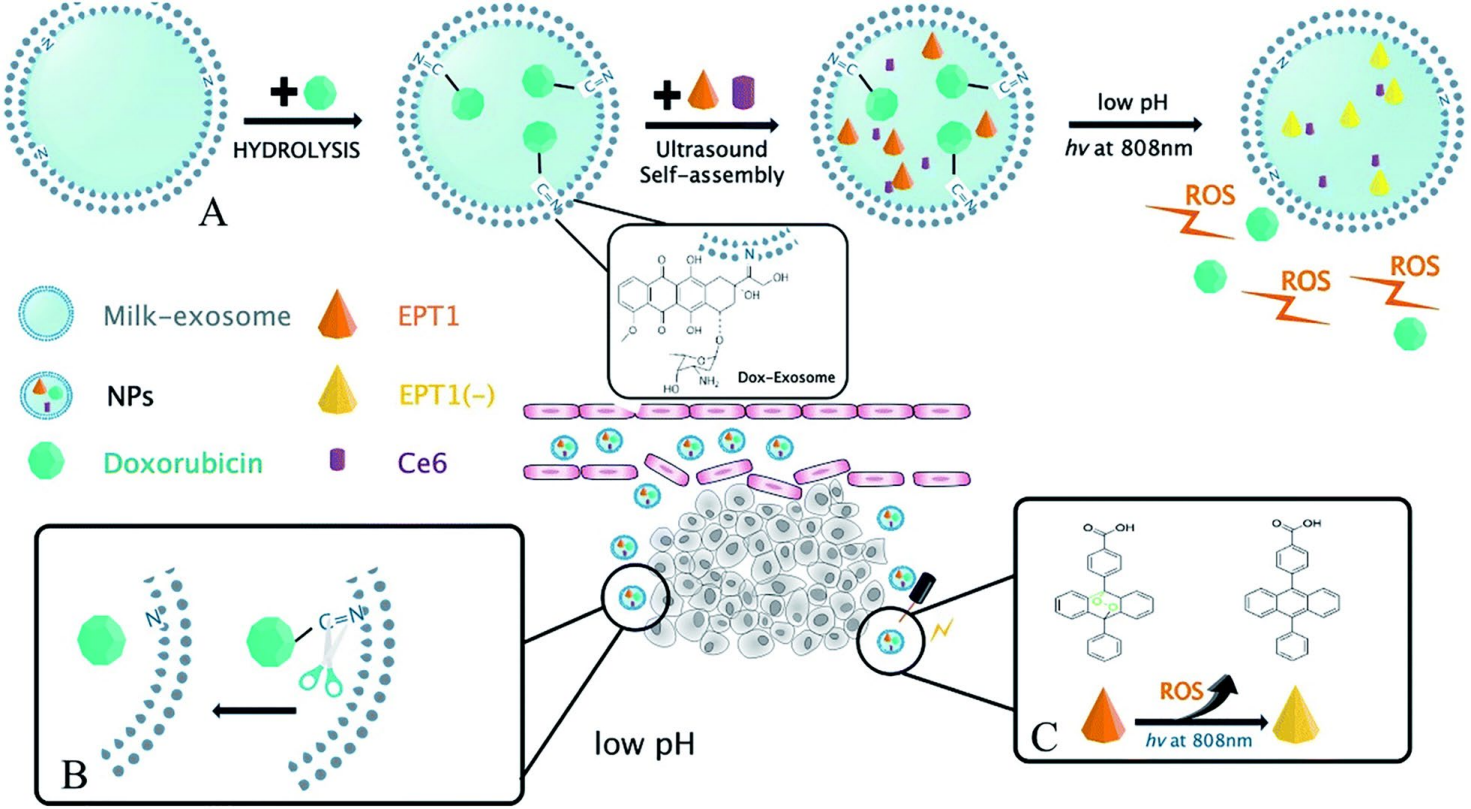
**A** Schematic illustration of the synthesis process for Exo@Dox–EPT1 (NPs). **B** Therapeutic mechanism of NPs under acidic tumor microenvironment. **C** PTT mechanism of NP under 808 nm near-IR light irradiation [116]

A large body of evidence suggests that engineered exosomes containing therapeutic agents can attenuate the oncogenic activity of human cancer cells, and therefore there is an urgent need to develop specific OSCC-targeted Exosomes (oct-Exosome). Hypoxia is a common feature of solid tumors and is associated with aggressiveness and poor patient prognosis. Li et al. found that exosomes derived from hypoxic oral squamous cell carcinoma cells increased migration and invasion of oral squamous cells by a HIF-1α and HIF-2α-dependent manner. And miRNA sequencing was performed on anorexia and hypoxic OSCC-derived exosomes, in which miR-21 was one of the most significantly upregulated miRNAs under hypoxic conditions. The results prompts further studies on the therapeutic value of exosome inhibition for cancer treatment [112]. Studies have shown that miRNA-34a has inhibitory effects on the proliferation, migration and invasion of OSCC. However, the lack of a safe and effective delivery system limits the clinical application of miRNA-34a in oral cancer therapy. Deng et al. loaded cholesterol-modified miRNA-34a into exosomes of HEK293T cells by co-incubation, it was finally taken up by oral squamous carcinoma cell. Exosomes loaded with miRNA-34a inhibited oral squamous carcinoma cell proliferation, migration and invasion by down-regulating SATB6 expression. It provides a new approach for the treatment of oral cancer [113]. Yutaro Kase et al. constructed exosomes of normal fibroblasts transfected with Epstein-Barr virus-inducible 3 (EBI3) cDNA by electroporation with siRNA for lymphocyte cytoplasmic protein 1 (LCP1) as engineered exosomes. The experiments showed that engineered exosomes stably and efficiently transferred siLCP1 into OSCC cells, the LCP1 was down-regulated in OSCC cells using engineering Exosomes, which resulted in in vitro and in vivo producing significant tumor suppressor effects [114]. Exosomes loaded with miR-155 inhibitors can reverse chemoresistance in oral cancer, thus providing an alternative therapeutic strategy for the treatment of refractory oral cancer patients [115]. Zhang et al. developed a pH/photosensitive drug system based on milk exosome for the treatment of oral small cell carcinoma. It is called exosome-doxorubicin-anthracene peroxide derivatives (Exo@Dox-EPT1, NPs).The main scheme of their study is depicted in Fig. 10. The milk exosome binds to doxorubicin, in addition, is loaded with endoperoxide and chlorine e6 (Ce6), which releases single-linear oxygen to kill cancer cells. The new milk exosome-based drug delivery system has been shown to be effective in the treatment of OSCC [116]. One study recently developed a therapeutic drug candidate exoASO-STAT6 carrying engineered exosome, which provides antisense oligonucleotides (ASOs) targeting signal transducer and activator of transcription 6 (STAT6). ASOs selectively silence STAT6 expression in TAM (tumor-associated macrophages), exoASO-STAT6 monotherapy produced an effective anti-tumor response, prompting clinical researchers to start investigating new cancer therapies as effective monotherapy candidates for other types of cancer, including OSCC [117].

## Conclusions and prospects

Considerable progress has been made in the field of exosomes over the past decade and we have gained a deeper understanding of their biological origin, molecular content and biological function. The role of exosomes in diagnosing and treating diseases of the oral and maxillofacial system has received considerable research attention, due to the potential role of exosomes as biomarkers and therapeutic agents. The use of exosomes as biomarkers and therapeutic agents in clinical applications has several advantages. The use of exosomes as diagnosis has the advantages of minimal invasiveness and wide availability in various body fluids. The diversity of exosome content provides a variety of diagnostic indicators that can improve the sensitivity and specificity of diagnosis. In the treatment of oral and maxillofacial diseases, exosomes are mediators of intercellular communication, transporting their contents to recipient cells and influencing oral disease progression by modulating host-associated immune responses, angiogenesis, drug resistance or invasive metastasis. Exosomes accommodate a variety of biomolecule types, which simultaneously exert different therapeutic mechanisms.

More importantly, due to the unique structure and physicochemical characteristics of exosomes, lead to the exosomes can be modified, and a large number of studies have shown that engineered exosomes can exhibit better diagnostic and therapeutic effects than nature exosomes. This paper we briefly discuss the engineering methods of exosomes, including modifying maternal cells, directly modifying exosomes, or biomaterial loading exosomes, focusing on the application of engineered exosomes in periodontitis, pulpitis, oral cancer, etc. Overall, engineered exosomes can exhibit better properties, such as stronger targeting, more active ingredients, and higher transport efficiency, which are not available in natural exosomes. But there is still a lot of room for improvement in the methods of exosome isolation and purification. Despite the current challenges, the idea of using engineered exosomes as a diagnostic and therapeutic tool for oral and maxillofacial diseases is promising, and the use of engineered exosomes is expected to be used more and more widely in oral and maxillofacial diseases in the near future.
